# IL-17A/F-Signaling Does Not Contribute to the Initial Phase of Mucosal Inflammation Triggered by *S*. Typhimurium

**DOI:** 10.1371/journal.pone.0013804

**Published:** 2010-11-23

**Authors:** Pascal Songhet, Manja Barthel, Till A. Röhn, Laurye Van Maele, Delphine Cayet, Jean-Claude Sirard, Martin Bachmann, Manfred Kopf, Wolf-Dietrich Hardt

**Affiliations:** 1 Institute of Microbiology, D-BIOL, ETH Zürich, Zürich, Switzerland; 2 Cytos Biotechnology AG, Schlieren, Switzerland; 3 Institut Pasteur de Lille, Center for Infection and Immunity of Lille, Institut National de la Santé et de la Recherche Médicale, U1019, CNRS, UMR 8204, Université Lille Nord de France, Lille, France; 4 Molecular Biomedicine, Institute of Integrative Biology, ETH Zürich, Zürich, Switzerland; Technical University Munich, Germany

## Abstract

*Salmonella* enterica subspecies 1 serovar Typhimurium (*S*. Typhimurium) causes diarrhea and acute inflammation of the intestinal mucosa. The pro-inflammatory cytokines IL-17A and IL-17F are strongly induced in the infected mucosa but their contribution in driving the tissue inflammation is not understood. We have used the streptomycin mouse model to analyze the role of IL-17A and IL-17F and their cognate receptor IL-17RA in *S*. Typhimurium enterocolitis. Neutralization of IL-17A and IL-17F did not affect mucosal inflammation triggered by infection or spread of *S*. Typhimurium to systemic sites by 48 h p.i. Similarly, *Il17ra^−/−^* mice did not display any reduction in infection or inflammation by 12 h p.i. The same results were obtained using *S*. Typhimurium variants infecting via the TTSS1 type III secretion system, the TTSS1 effector SipA or the TTSS1 effector SopE. Moreover, the expression pattern of 45 genes encoding chemokines/cytokines (including CXCL1, CXCL2, IL-17A, IL-17F, IL-1α, IL-1β, IFNγ, CXCL-10, CXCL-9, IL-6, CCL3, CCL4) and antibacterial molecules was not affected by *Il17ra* deficiency by 12 h p.i. Thus, in spite of the strong increase in *Il17a*/*Il17f* mRNA in the infected mucosa, IL-17RA signaling seems to be dispensable for eliciting the acute disease. Future work will have to address whether this is attributable to redundancy in the cytokine signaling network.

## Introduction

*S*. Typhimurium is a Gram-negative enteropathogen and a frequent cause of diarrhea. After ingestion, the bacteria travel through the alimentary tract, manipulate cells of the intestinal mucosa and thereby elicit the disease which is characterized by fluid accumulation in the gut lumen and gut tissue inflammation. We are just beginning to understand the complex molecular interplay between the pathogen and the host which leads to disease.

*S*. Typhimurium induced gut inflammation has been studied in bovine, murine and primate animal models. In all three models, it involves epithelial damage and PMN influx and similar chemokine/cytokine induction profiles. This includes the chemokines/cytokines CXCL1, CXCL2, IL-1α, IL-1β, IFNγ, IL-17A, IL-17F, CXCL-10, CXCL-9, IL-6, CCL3, CCL4 some of which are strong phagocyte chemo-attractants, as well as molecules implicated in anti-microbial defense like Nos2, lipocalin 2 and antimicrobial peptides ([1], [2], [3], own unpublished data). This similarity implies that the molecular mechanisms driving the disease are equivalent in all three animal models.

So far, the detailed mechanism for the induction of mucosal inflammation by *Salmonella* spp. remains unclear. Most likely, initial signals are produced by the few cells of the mucosa which become directly infected as well as by recognition of microbe associated molecular patterns (MAMP; e.g. lipidA, flagellin) released by the invading pathogen [4], [5], [6], [7], [8], [9], [10]. Several lines of evidence imply a key role for IL-17 signaling in *S*. Typhimurium diarrhea. a) IL-17 signaling is implicated in several cases of mucosal infection or inflammation, i.e. *Klebsiella pneumoniae* lung infections [11], [12], *Mycoplasma pneumoniae* infection [13], allergic airway inflammation [14], *Helicobacter hepaticus* colitis [15] and DSS-induced colitis [16]. Furthermore, segmented filamentous bacteria (SFB) induce a Th17 response that acts protective against intestinal pathogen *Citrobacter rodentium* [17]. Thus, by analogy IL-17 signaling might also contribute to mucosal *S*. Typhimurium infection. b) IL-17A and IL-17F are strongly induced in the *Salmonella*-infected gut ([1], [18], [19], [20], own unpublished data). *Il17ra*, which encodes a key subunit of a receptor binding IL-17A and IL-17F, is expressed on many cell types including cells of the intestinal mucosa (reviewed in [21]). c) *S*. Typhimurium-induced gut inflammation was attenuated in *Il17ra^−/−^* mice by day 2 post infection [19]. However, the specific roles of IL-17A and IL-17F and their downstream targets in *S*. Typhimurium-induced mucosal inflammation have remained poorly defined.

*S*. Typhimurium expresses multiple virulence factors capable of eliciting gut inflammation. The type III secretion systems encoded in *Salmonella* pathogenicity islands-1 and -2 (termed TTSS1 and TTSS2 in this paper) are prominent factors [10], [22]. TTSS1 triggers the inflammation during the first day of infection even in the absence of TTSS2 [23], while TTSS2 contributes to mucosal disease at later time points [10], [22]. To characterize the contribution of TTSS1 to inflammation during the first hours of infection and to exclude any potential influence of TTSS2, one can use the mutant *S*.Tm* which lacks TTSS2 (SL1344, *sseD::aphT*; [10], [22]). Similarly, the respective contribution of the TTSS1 “effector proteins” SipA and SopE can be studied using appropriate site directed mutants: SipA-dependent caspase-1 independent inflammation, using *S*.Tm^SipA^ (SL1344, *sseD::aphT sopE sopB sopE2*; [9], [10]) and SopE-dependent caspase-1 dependent inflammation using *S*.Tm^SopE^ (SL1344, *sseD::aphT sipA sopB sopE2*; [9], [10]). Strains lacking TTSS1 and TTSS2 (*S*.Tm^avir^; SL1344 *ΔinvG sseD::aphT*; [24]) do not elicit gut inflammation. These strains should provide sensitive tools for studying how IL-17 responses are triggered and how they drive the disease.

In this manuscript, we have studied the role of IL-17A and IL-17F-signaling in the initial phase of the *Salmonella* gut infection. We analyzed the effects of IL-17A or -F neutralization and compared disease pathology and cytokine profiles elicited in wt mice and isogenic *Il17ra^−/−^* littermates. Gut inflammation and pronounced cytokine induction was observed in all animals infected with virulent *S*. Typhimurium strains. However, neither disease pathology nor cytokine induction profiles were affected by cytokine neutralization or *Il17ra* deficiency by 12 h p.i.. Thus, IL-17A and IL-17F signaling does not seem to affect the initial phase of acute mucosal disease.

## Materials and Methods

### Ethics Statement

All animals were handled in strict accordance with good animal practice as defined by the relevant national and/or local animal welfare bodies, and all animal work was approved by the appropriate committee (Kantonales Veterinäramt Zürich, Zürich Switzerland, licence number 201/2007).

### Mice

All mice were bred and kept specified pathogen free in individually ventilated cages (RCHCI, ETH Zürich and Biosupport, Schlieren). *Il17ra^−/−^* mice (C57BL/6 N5 backcross) ([25]; 5 generations in C57BL/6 background) were kindly provided by Amgen and backcrossed for one more generation onto C57BL/6 (Charles River). Mice were genotyped by PCR using the following oligonucleotides: KS35.20: AGCTGCTGTTAGCACTTTGC; TGX53.18: CTTGTGTAGCGCCAAGTG; KS37.19: CGTACGCACACACTCTCGA. *Il17ra^+/−^* and *Il17ra^−/−^* (C57BL/6 N6 backcross) littermates were used for experiments together with C57BL/6 mice from our colony as positive controls. Mice of all groups were sex and age matched (8–10 weeks old).

Mice were pretreated with streptomycin (1 dose, 20 mg/animal, by gavage) and infected with 5×10^7^ cfu by gavage 24 h later [26], [27]. Live bacterial loads (colony forming units, cfu) in mesenteric lymph nodes (mLN), spleen and the cecal content were determined by plating [27].

### Bacterial strains

All *S*. Typhimurium strains are isogenic derivatives of SL1344 [28]. *S*.Tm* (SL1344, *sseD::aphT*; M556), *S*.Tm^SipA^ (SL1344, *sseD::aphT sopE sopB sopE2*; M716), *S*.Tm^SopE^ (SL1344, *sseD::aphT sipA sopB sopE2*; M717) and *S*.Tm^avir^ (SL1344, *ΔinvG sseD::aphT*; M557) have been described, recently [9], [24]. The strains were grown for 12 h at 37°C in LB (0.3 M NaCl) and sub-cultivated for 4 h as described [24].

### Histopathology

HE-stained cecum cryosections were scored as described, evaluating submucosal edema, PMN infiltration, goblet cells and epithelial damage yielding a total score of 0–13 points [26].

### Vaccination of mice using IL-17A-VLP and IL-17F-VLP

VLP-based IL-17 vaccines were generated as described recently [29]. Briefly, murine IL-17A (aa 26–158) and murine IL-17F (aa 29–161) flanked by an N-terminal hexa-histidine tag and a C-terminal linker sequence consisting of five glycine residues and one cysteine residue were recombinantly expressed in *E.coli* strain BL21. Purified and refolded cytokines were then covalently linked to VLPs of the bacteriophage Qβ using the heterobifunctional crosslinker SMPH as described [29], resulting in VLPs displaying either the cytokine IL-17A (IL-17A-VLPs) or the cytokine IL-17F (IL-17F-VLPs).

Mice were immunized thrice in 10 day intervals by s.c. injection of 50 µg of either VLPs alone, IL-17A-VLPs, IL-17F-VLPs or a mixture of both. Two weeks after the last immunization sera of immunized mice were analyzed for presence of αIL-17A or αIL-17F IgGs by ELISA as described previously.

### Quantitative RT-PCR

The cecum tissue was excised, washed in cold PBS, placed in 600 µl RNAlater-buffer (RNeasy Mini Kit, Qiagen), stored for 2 h on 4°C and subsequently frozen. Total RNA was extracted using a Nucleospin RNA II kit (Macherey Nagel, Germany) and reverse-transcribed using the High-Capacity cDNA Archive Kit (Applied Biosystems, USA). The resulting cDNA was amplified using TaqMan assays in the Taqman Low Density Array (TLDA) format (Applied Biosystems). Analysis was carried out using Real Time StatMiner software from Integromics. Relative mRNA levels (2^−ΔΔCt^) were determined by comparing (a) the PCR cycle thresholds (Ct) for 45 genes of interest and the 3–4 house-keeping genes *18S, Actb, and Gapdh* (or *B2m*) (ΔCt) and (b) ΔCt values for treated and control groups (ΔΔCt). In all experiments, Ct upper limit was fixed to 33 cycles.

For analysis of *Il17ra* expression, RNA was processed using the RNeasy mini kit (Qiagen), the RNase-Free DNase kit (Qiagen), M-MLV reverse Transcriptase RNase H Minus (Promega) and RNasin (Promega). qPCR analysis of *Il17ra* (5′- AGTGTTTCCTCTACCCAGCAC-3′ and 5′-GAAAACCGCCACCGCTTAC-3′)[30] and GAPDH (5′-GGCTGCCCAGAACATCATCCCTGCAT-3′ and 5′-ACGTCAGATCCACGACGGACACATTGG-3′) was performed using the SensiMix Plus SYBR Green kit (Peqlab Biotechnologie GMBH, Germany). Relative mRNA levels (2^−ΔΔCt^) were determined by comparing (a) the PCR cycle thresholds (Ct) for *Il17ra* and *Gapdh* (ΔCt) and (b) ΔCt values for treated and control groups (ΔΔCt). The Ct upper limit was fixed to 33 cycles. Cycling parameters were 94°C (15 s), 60°C (30 s), 72°C (30 s) in a RotorGene 3000 cycler (Corbett Research, Cambridgeshire, UK).

### Statistical analysis

Statistical analysis of the animal experiments was performed using the exact Mann-Whitney U test. To perform statistical analysis, minimal detectable bacterial colonization levels (CFU) were set to 10 CFU/mLN, 20 CFU/spleen, 60 CFU/liver, 10 CFU/g cecum content, in cases where no bacteria were detected by plating. A *P* value of <0.05 (two tailed) was considered to be statistically significant.

The Limma test with Bonferroni multiple hypothesis testing was used for high throughput PCR analyses with Taqman Low Density Arrays. For the RT-PCR analysis of the *Il17ra* expression in the cecum, the unpaired t-test (two-tailed) has been used. Cecal pathology of cytokine vaccinated mice has been analyzed by the exact Mann-Whitney U test as well as ANOVA with Bonferroni post test.

## Results

### Antibody-mediated neutralization of IL-17A and IL-17F did not affect *S*. Typhimurium enterocolitis

First, we employed a neutralization strategy to analyze the contribution of IL-17A and/or IL-17F in *S*. Typhimurium enterocolitis. We neutralized endogenous IL-17A and IL-17F by vaccination of C57BL/6 mice with virus-like particles coupled to murine IL-17A (IL-17A-VLP) or IL-17F (IL-17F-VLP) proteins as described previously [29], [31]. This earlier work had established that IL-17 cytokine vaccination is capable of alleviating IL-17 driven disease in two different models of chronic inflammatory disease *in vivo*. The IL-17A-VLP/IL-17F-VLP vaccination strategy is of advantage, as it avoids possible artefacts attributable to ontogenetic defects in immune system maturation which may be observed in knockout mice. In addition, all mice come from the same colony which circumvents any variations emanating from microbiota composition which may affect the results of the infection experiments (Stecher and Hardt, unpublished observation).

**Figure 1:**
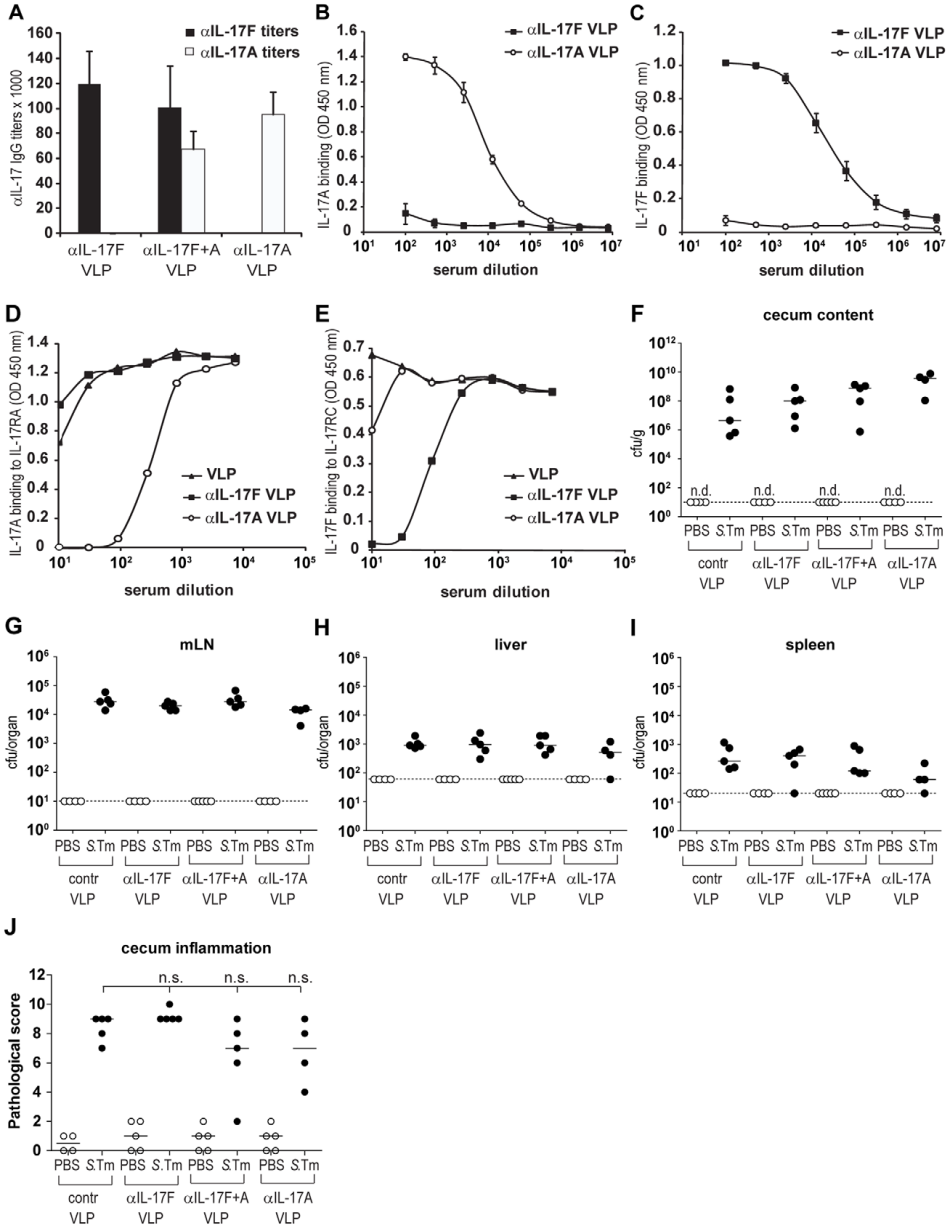
Effect of IL-17A and/or F immunization on the *S.*Typhimurium infection. C57BL/6 mice were vaccinated three times (50 µg each, s.c.) with IL-17A-VLP, IL-17F-VLP, either reagents or unconjugated control VLPs. A–C) αIL-17A and αIL-17F titers were analyzed two weeks after the last immunization by ELISA and compared to unconjugated-VLP immunized controls (B,C; OD450 nm values +/− SEM). D,E) Neutralization was tested by ELISA. D) ELISA plates were coated with 1 µg/ml IL-17RA and binding of 10 ng/ml biotinylated IL-17A to IL-17RA was tested in the presence of serum of mice immunized with IL-17A-VLPs, IL-17F-VLPs or VLPs alone (OD450 nm values +/− SEM). E) ELISA plates were coated with 1 µg/ml IL-17RC and binding of 200 ng/ml biotinylated IL-17F to IL-17RC was tested in the presence of serum of mice immunized with IL-17A-VLPs, IL-17F-VLPs or VLPs alone; OD450 nm values +/− SEM. F–J) Subsequent analysis in *S*. Typhimurium challenge infections. Animals were pretreated with streptomycin and infected for 2 days with wt *S*. Typhimurium. PBS immunization and PBS treatment served as control. We analyzed colonization levels in the gut lumen (F), the mLN (G), the liver (H) and the spleen (I), as well as the degree of inflammation of the cecum mucosa (J). Comparison with the control VLPs immunized mice did not reveal any significant differences in any of the parameters of the infection, analyzed (J: Mann-Whitney U test was not significant as well as ANOVA with Bonferroni (p = 0.0841)). Stippled line: minimal detectable value.

C57BL/6 mice (n = 5 mice per group) were vaccinated with either IL-17A-VLP, IL-17F-VLP, both reagents or unconjugated control VLPs (Materials and Methods). Two weeks after immunization, αIL-17A and αIL-17F serum IgG responses were verified by ELISA (Fig. 1A–C) and by inhibiting the binding of IL-17A to IL-17RA or IL-17F to IL-17RC (Fig. 1D,E). Then, the mice were pretreated with streptomycin and infected by gavage with wild type *S*. Typhimurium (*S*.Tm, 5*10^7^ cfu). Bacterial loads in the gut lumen, mLNs, spleens and livers showed no significant differences comparing non-vaccinated and vaccinated mice (p≥0.05; Fig. 1F–I) two days post infection. Moreover, histopathological analysis revealed comparable inflammation of the cecum mucosa (p≥0.05; Fig. 1J). These results demonstrated that neutralization of IL-17A and/or IL-17F did not affect the course of *Salmonella* enterocolitis during the first two days of infection in the streptomycin mouse model. Similar results were obtained in IL-17A knockout mice infected with wildtype *S*. Tm (data not shown).

Taken together, these results suggested that IL-17A and IL-17F may play a limited (if any) role in *S*. Typhimurium-induced gut inflammation.

### *Il17ra^−/−^* mice showed normal levels of *S*.Typhimurium-induced gut inflammation at 12 h p.i

In a second series of experiments, to assess the roles of IL-17A and IL-17F in *Salmonella* enterocolitis, we studied mice lacking IL-17RA, which binds both IL-17A and IL-17F. *Il17ra^−/−^* (C57BL/6 N6; [25]) and control *Il17ra^+/−^* littermates or C57BL/6 wild-type mice were infected with *S*.Tm* or isogenic derivatives thereof, i.e. *S*.Tm^SipA^, *S*.Tm^SopE^ or *S*.Tm^avir^ (Materials and Methods). *S*.Tm* is known to elicit profound gut inflammation via TTSS1 which is equivalent to that by wt *S*. Typhimurium, but eliminates any possible contributions of TTSS2 [9], [23], [24], [32]. Therefore, *S*.Tm* should provide a more sensitive readout than wt *S*.Tm for any effects of IL-17RA on *Salmonella* enterocolitis. *S*.Tm^SipA^ and *S*.Tm^SopE^ rely on SipA or SopE respectively for eliciting inflammation [9], [24]. This may reduce redundancy in the pro-inflammatory signaling pathways and should reveal even very subtle susceptibility phenotypes of the knockout mice. The control strain *S*.Tm^avir^ does not elicit gut inflammation [10]. All animals were pretreated with streptomycin and infected for 12 h with 5×10^7^ cfu (by gavage; n = 3–7 mice per group) of the indicated strain. By 12 h post infection, wild type mice have already mounted gut inflammation (Songhet, Barthel, Hardt, unpublished). Any delays in mounting gut inflammation in the knockout mice should be detectable with high sensitivity at this time point. After 12 h, the animals were sacrificed, the pathogen loads in the cecum lumen, the mLN and the spleens were analyzed and we removed cecum tissue samples for histopathological analysis and for RNA extraction.

**Figure 2:**
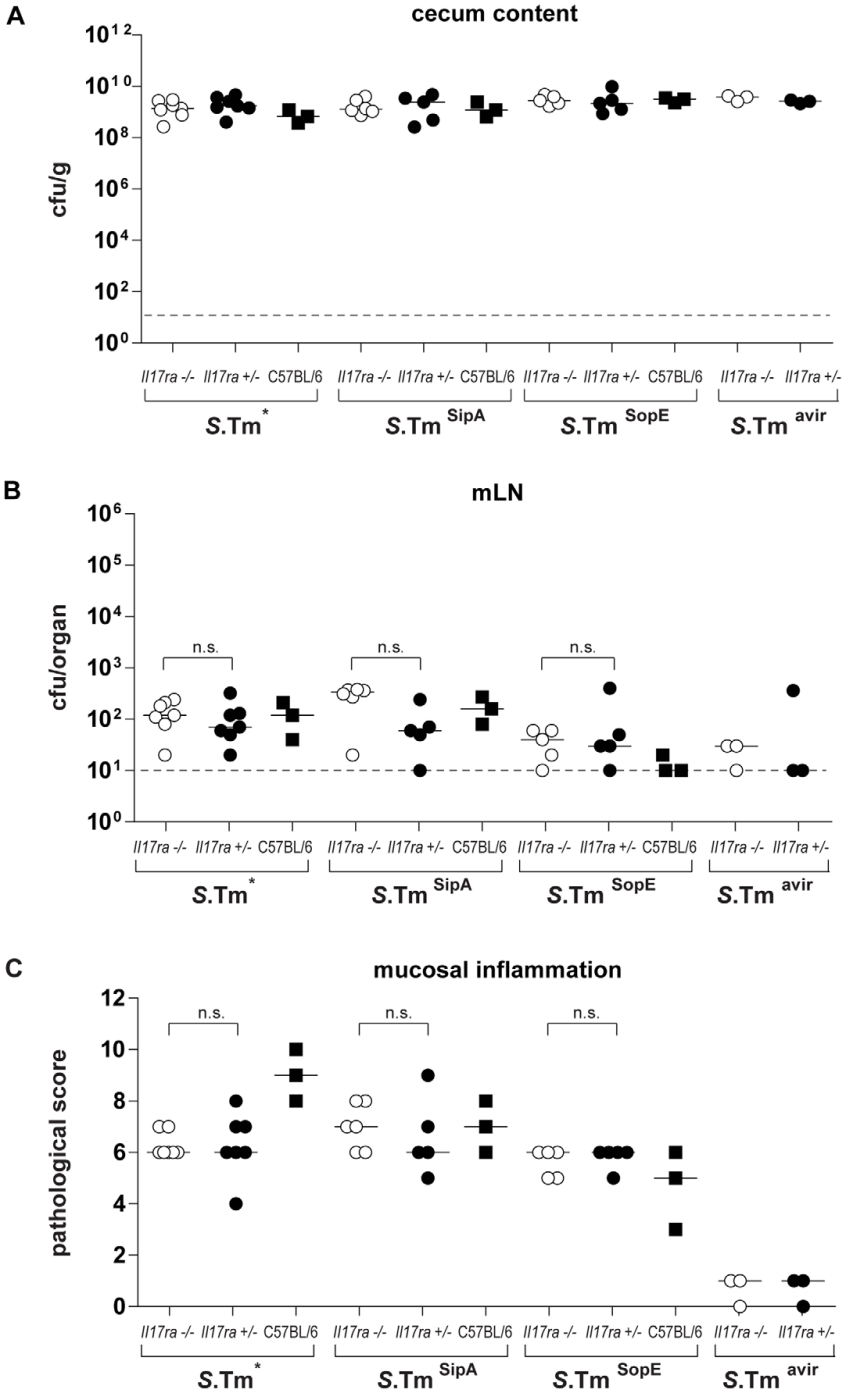
*S*.Typhimurium infection of *Il17ra^−/−^* mice. *Il17ra^−/−^* mice, *Il17ra^+/−^* littermates and C57BL/6 control mice were pretreated with streptomycin and infected for 12 hours with *S*.Tm* or isogenic derivatives *S*.Tm^SipA^, *S*.Tm^SopE^, *S*.Tm^avir^ (Material and Methods). We analyzed colonization levels in the gut lumen (A) and the mLN (B), as well as the degree of inflammation of the cecum mucosa (C). n.s.: No significant difference between *Il17ra^−/−^* mice and *Il17ra^+/−^* littermates. Strippled line: minimal detectable value.

All bacterial strains efficiently colonized the cecum lumen in all mice analyzed (Fig. 2A). Spread to mLN was not significantly affected in any of the groups, indicating that dissemination to deeper sites of the GALT was not affected in the *Il17ra^−/−^* mice at this early stage of the infection (p≥0.05; Fig. 2B). Moreover, we did not detect spread to spleens and livers in the *Il17ra^−/−^* (data not shown). However, as these systemic sites are normally not colonized by the pathogen at these early time points, we cannot draw conclusions about the role of IL-17RA-deficiency in systemic pathogen spread.

**Figure 3:**
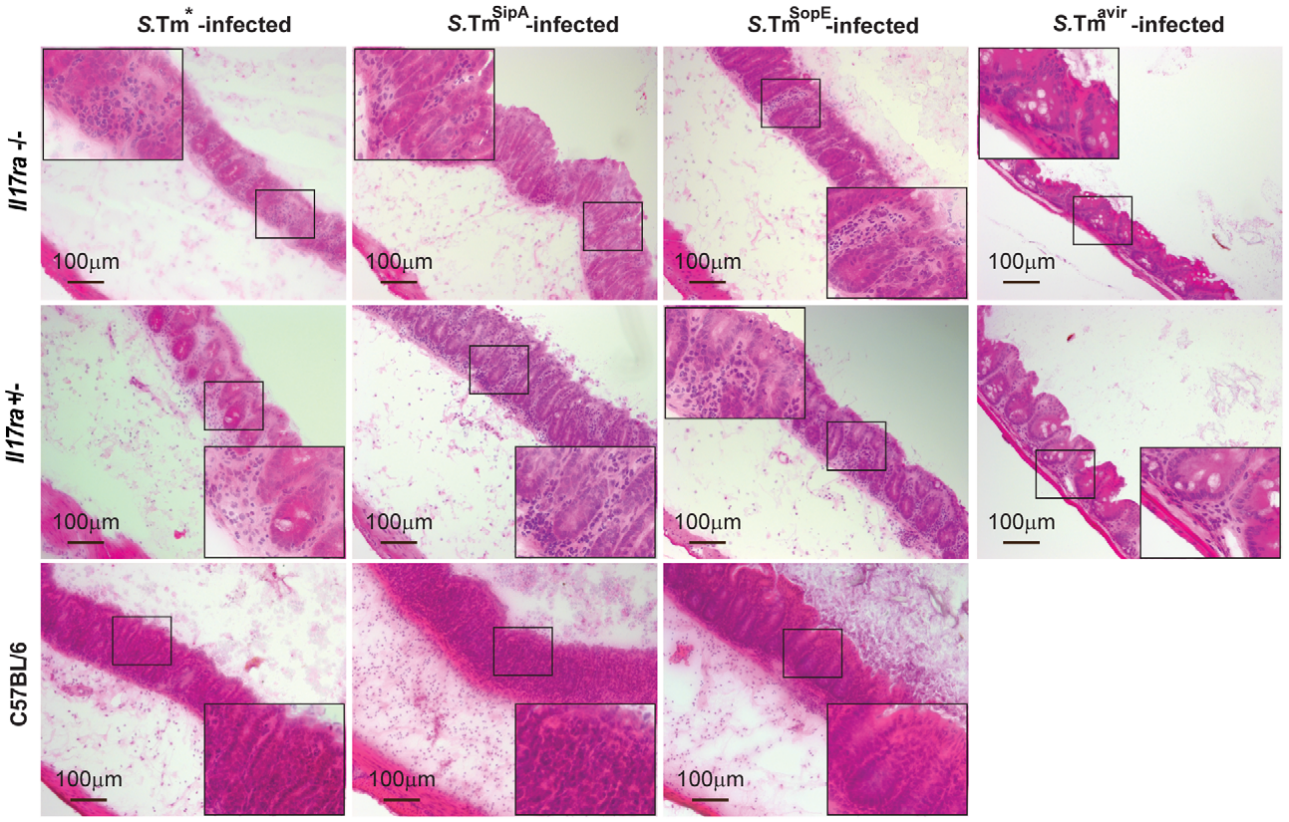
Mucosal inflammation in *S*.Typhimurium infected *Il17ra ^−/−^* mice. Cryosections of *Il17ra ^+/−^*, *Il17ra^−/−^* and C57BL/6 mice infected with *S*.Tm*, *S*.Tm^SipA^ and *S*.Tm^SopE^ for 12hpi were stained with HE. Representative animals were chosen from the experiment shown in Fig. 2. Bar  = 100 µm.

As expected, *S*.Tm*, *S*.Tm^SipA^ and *S*.Tm^SopE^ triggered overt inflammation in the cecal mucosa of the IL-17A/F-signaling proficient *Il17ra^+/−^* littermates and the wt C57BL/6 control mice (Fig. 2C, Fig. 3). Surprisingly, *S*.Tm*, *S*.Tm^SipA^ and *S*.Tm^SopE^ triggered equivalent levels of gut inflammation in the *Il17ra^−/−^* and in the *Il17ra^+/−^* littermates (p≥0.05; Fig. 2C, Fig. 3). Thus, IL-17RA signaling was dispensable for mounting mucosal inflammation by 12h p.i..

### Cytokine profiles were not altered in the cecum tissue of *S*.Typhimurium-infected *Il17ra^−/−^* mice at 12 h p.i

**Figure 4:**
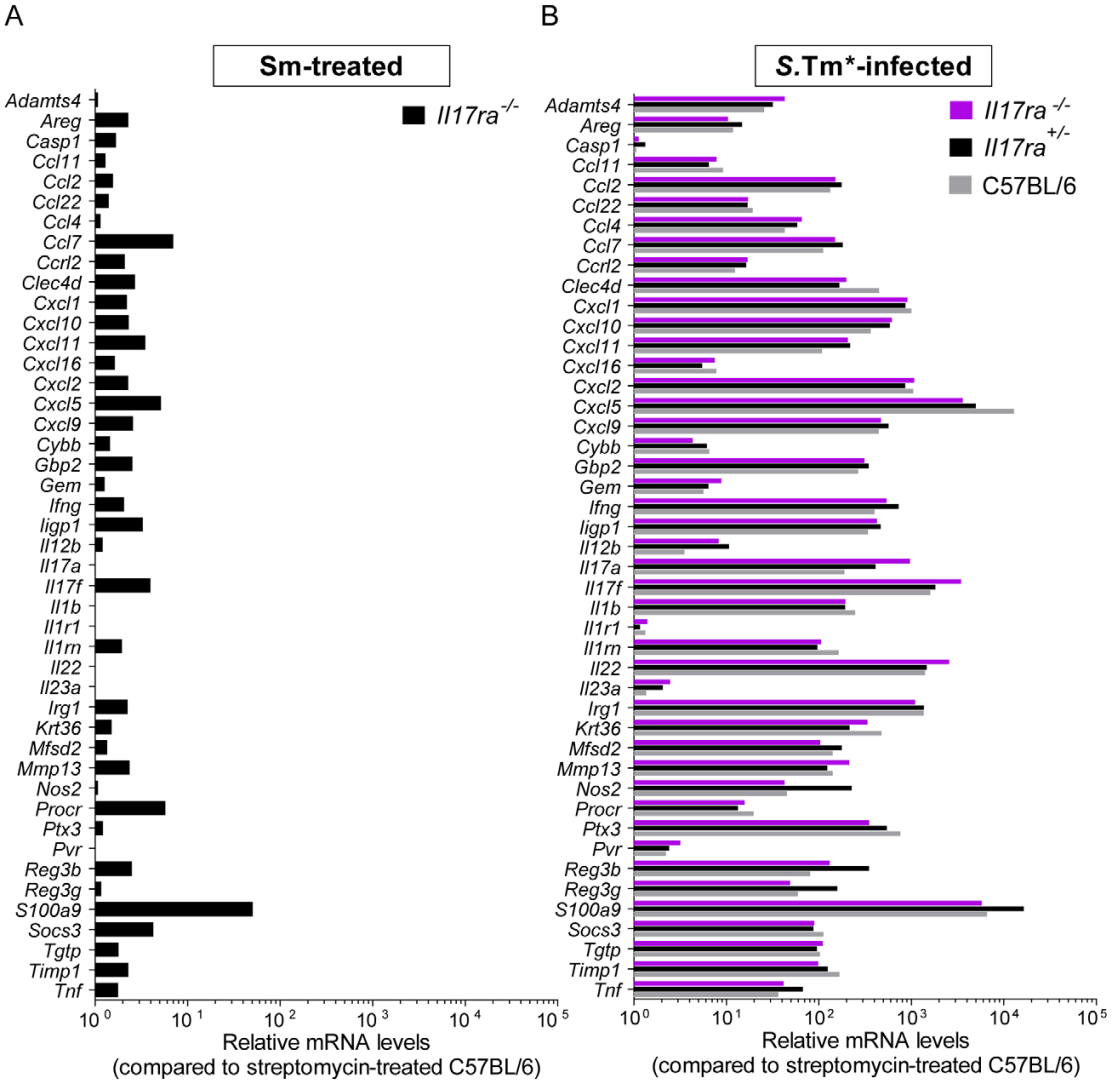
Cytokine expression profile of *S*.Tm^*^-infected *Il17ra^+/−^*, *Il17ra^−/−^*and C57BL/6 mice. *Salmonella*-infected animals (n = 3) from experiment shown in Fig. 2 were used for analysis of gene expression signature. Cecum samples from *Il17ra^−/−^, Il17ra^+/−^,* and C57BL/6 mice 12 h p.i. with *S*.Tm*, *S*.Tm^SipA^ or *S*.Tm^SopE^, were taken for RNA isolation and RT-PCR (B–D). C57BL/6 mice, only treated with streptomycin, were used as a control, not differing significantly from *Il17ra^−/−^* (A). 45 genes related to inflammation and defense, especially in IL-17-mediated responses were used. The analysis was performed using RealTime StatMiner Software; mRNA levels were normalized to 3 house-keeping genes: *Actb, Gapdh* and *18S* (ΔCt). Relative quantification was determined by the ΔΔCt method after normalization to streptomycin-treated C57BL/6 animals. Statistical analysis was done using the Limma test with Bonferroni correction (B). All results for *S*.Tm^*^-infection did not differ significantly between *Il17ra^−/−^* and *Il17ra^+/−^* mice (p≥0.05).

**Figure 5:**
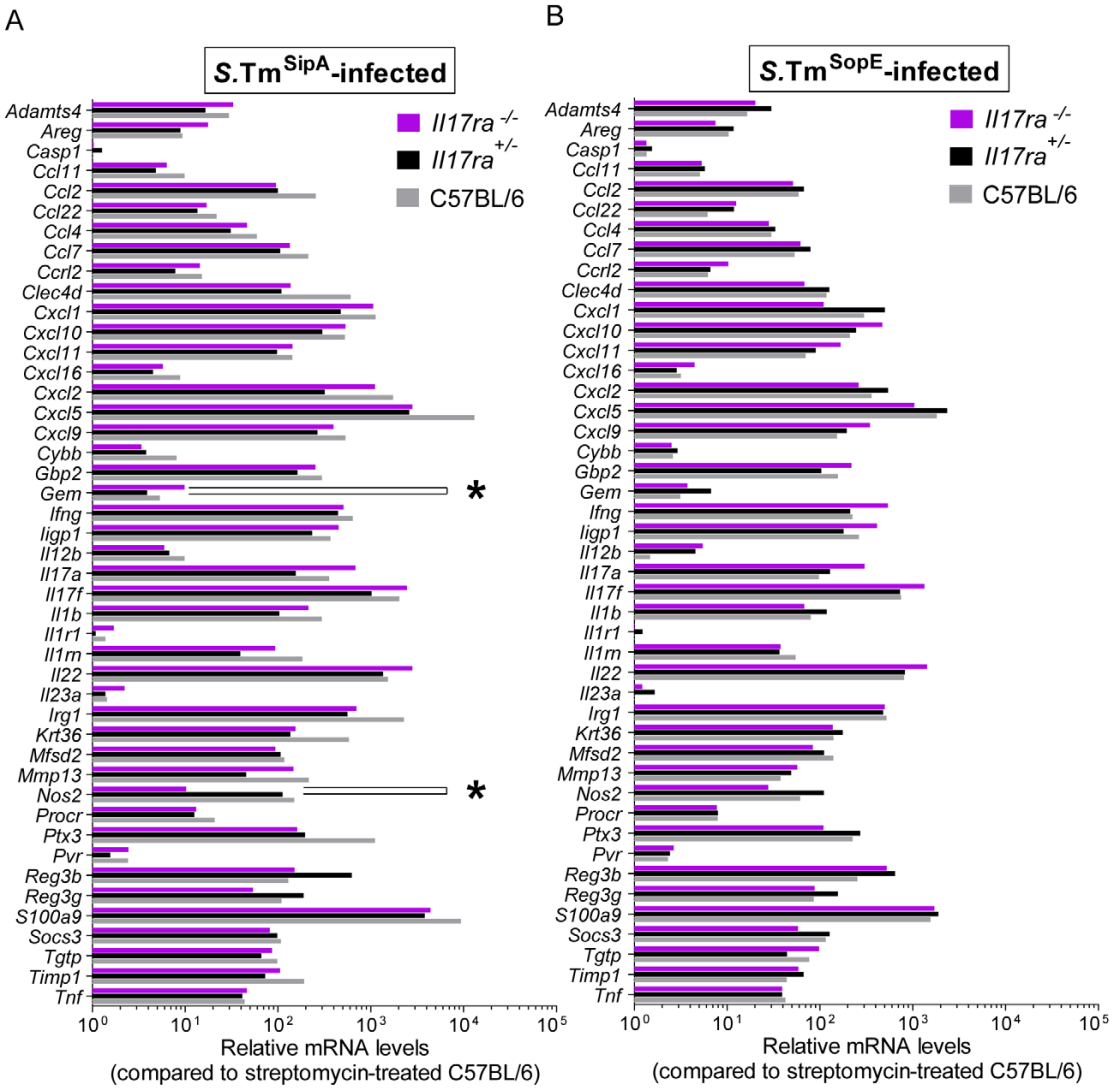
Cytokine expression profile of *S*.Tm^SipA^- and *S*.Tm^SopE^-infected *Il17ra^+/−^*, *Il17ra^−/−^* and C57BL/6 mice. *Salmonella*-infected animals (n = 3) from experiment shown in Fig. 2 were used for analysis of gene expression signature. Cecum samples from *Il17ra^−/−^, Il17ra^+/−^,* and C57BL/6 mice 12 h p.i. with *S*.Tm^SipA^ or *S*.Tm^SopE^, were taken for RNA isolation and RT-PCR (A–B). 45 genes related to inflammation and defense, especially in IL-17-mediated responses were used. The analysis was performed using RealTime StatMiner Software; mRNA levels were normalized to 3 house-keeping genes: *Actb, Gapdh* and *18S* (ΔCt). Relative quantification was determined by the ΔΔCt method after normalization to streptomycin-treated C57BL/6 animals. Statistical analysis was done using the Limma test with Bonferroni correction (A–B). All results presented in panels A and B did not differ significantly between *Il17ra^−/−^* and *Il17ra^+/−^* mice (p≥0.05) with the exception of *Gem* and *Nos2* in panel A (*: p<0.05).

In order to analyze how *Il17ra* deficiency affected early immune responses, we have performed quantitative RT-PCR analysis using Taqman Low Density Arrays on cecum tissue samples from *Il17ra^−/−^*, *Il17ra^+/−^* and C57BL/6 mice. We determined the relative mRNA levels for a set of 45 genes encoding immune mediators, including IL-17A and IL-17F, as well as IL-22, CXCL1, CXCL2, IL-23 p19, IL-12 p40, IFNγ and TNFα. First, we found that steady-state levels of mRNA are similar in C57BL/6 and the IL-17RA-deficient mice (Fig. 4A). In line with previous work on a later stage of the infection (day 2 p.i.; [1], [18], [19]; own unpublished data), *S*.Tm* dramatically induced the expression of *Il17a* and *Il17f* as well as a large array of pro-inflammatory cytokines and innate defense molecules already by 12 h p.i. (Fig. 4B, grey bars; incl. antimicrobial peptides RegIIIβ, Reg3γ, S100A9, inducible nitric oxide synthase, or the pattern recognition pentraxin PTX3). This also included several genes known to be regulated or co-regulated by IL-17 cytokines (CXCL1, CXCL2, CXCL5, CCL2, CCL7 and S100A9; [2], [33]). Surprisingly, the gene expression profiles of the *S*.Tm* infected *Il17ra^−/−^* mice did not differ significantly from those of *Il17ra^+/−^* littermates or the wt C57BL/6 controls, including CXCL1, CXCL2, CXCL5, CCL2, CCL7 and S100A9 (p≥0.05 for all cytokines; *Il17ra^−/−^* vs. *Il17ra^+/−^;* Fig. 4B). The same was observed for the mice infected with either *S*.Tm^SipA^ or *S*.Tm^SopE^ (Fig. 5A,B). Slightly altered expression of two genes in *S*.Tm^SipA^ infected mice was the only exception (Gem, Nos2; p<0.05; Fig. 5A). Future work will have to address the reasons for this. Nevertheless, overall these data demonstrated that IL-17RA deficiency did not affect inflammatory pathology and mucosal chemokine/cytokine induction at 12 h post infection.

### *Il17ra* is expressed by the intestinal mucosa

**Figure 6:**
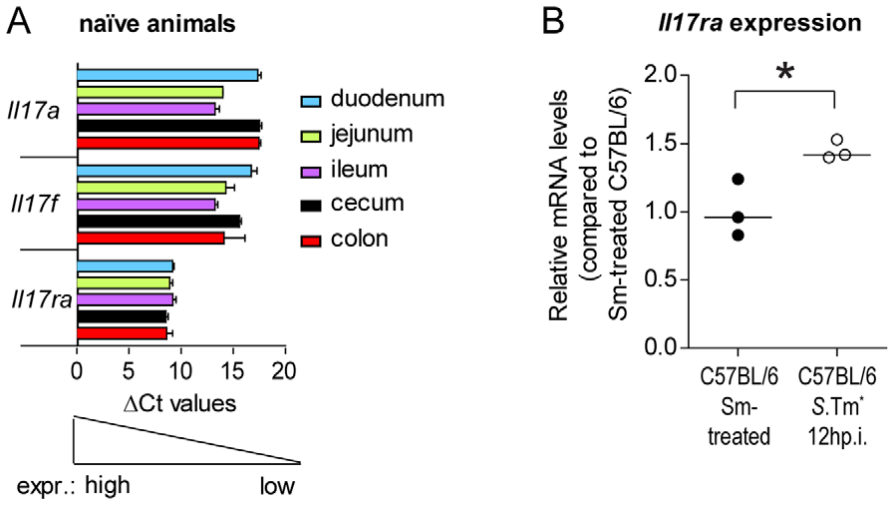
*Il17ra* expression in the gut of control and *S*.Tm^*^-infected C57BL/6 mice. (A) *Il17a*, *Il17f* and *Il17ra* expression in the intestinal tissues of naïve mice (n = 3 per group). Relative levels of mRNA in 20 ng total RNA were normalized to *house keeping genes*. ΔCt values do not strictly correlate to the difference in gene expression between various genes. However, ΔCt values <19 represent detectable levels of expression, demonstrating thereby that all three genes are expressed in the various tissue segments. (B) Induction of *Il17ra* expression in *Salmonella*-infected animals. *Salmonella*-infected animals (n = 3; 12 h p.i. with *S*.Tm*; open circles) were from the experiment shown in Fig. 2. Non-infected, stereptomycin pretreated C57BL/6 mice (n = 3; black circles) served as a contol. Samples of gut tissues (A, as indicated; B, cecum tissue) were taken for RNA isolation and mRNA levels were analyzed by real time PCR. *S*.Tm^*^ infected C57BL/6 mice showed a significant up-regulation of *Il17ra* in comparision to non-infected C57BL/6 mice (p<0.05; t-test unpaired, two-tailed). Sm: Streptomycin. Line: median.

Finally, we have verified that *Il17ra* is indeed expressed in the gut. For this purpose, we recovered tissue samples from the duodenum, jejunum, ileum, cecum and colon of naïve C57BL/6 mice and analyzed *Il17ra* mRNA levels by real time PCR. *Il17a* and *Il17f* served as controls. These data verified that the *Il17ra* gene is expressed to detectable levels in all parts of the naïve gut (Fig. 6A). In a second approach, we analyzed *Il17ra* induction during infection. 12 hours after infection with *S*.Tm*, the *Il17ra* expression in cecum tissues was slightly, but significantly higher than in streptomycin pretreated non-infected controls (Fig. 6B). Thus, *Il17ra* is clearly expressed in the naïve and in the infected intestinal mucosa.

## Discussion

*Salmonella* diarrhea involves the elicitation of acute mucosal inflammation within 6–10 h post infection. The acute mucosal response includes the transcriptional up-regulation of more than 100 genes encoding pro-inflammatory cytokines, antimicrobial defenses and cytokines initiating adaptive immune responses [1], [18], [19], [20]. It is assumed that initial signals emanate from those few mucosal cells which become infected in the initial phase of the infection, and that these initial signals are amplified subsequently in order to mount a tissue-wide defense. The induced genes have been identified, but their role in the disease process has remained poorly defined. Here, we have analyzed the role of IL-17 signaling. Levels of IL-17A and IL-17F encoding mRNA are strongly elevated in the *S*. Typhimurium infected cecum mucosa [1], [19]. We found that cytokine-neutralization of IL-17A and/or F or a genetic IL-17RA-deficiency did not affect *S*. Typhimurium colitis, i.e. pathogen colonization of the mLN, degree of intestinal inflammation and the patterns of cytokines induced by 12 h p.i.

Earlier work had assessed the role of IL-17 signaling at day 2 p.i. with *S*. Typhimurium 14028 [19]. This publication had employed the same streptomycin mouse model [26] as we have used in our study and found that *Il17ra^−/−^* mice failed to mount an efficient mucosal response (i.e. neutrophil influx, KC/CXCL1 induction) and had an increased susceptibility to systemic spread of the pathogen by day 2 p.i. [19]. This may suggest that IL-17 signalling affects disease only after the initial 12 h of the infection. Nevertheless, our IL-17A-VLP/IL-17F-VLP vaccinated mice were analysed at day 2 p.i.. In this experimental system, we did not observe disease phenotypes attributable to the anti-cytokine vaccination. However, we could not precisely control the degree of functional IL-17 cytokine neutralization within the gut tissues of the infected animals. Therefore, the lack of significant effects on gut pathology might be attributable to incomplete neutralization. Nevertheless, our data on IL-17 cytokine neutralization is of significant interest for assessing the possible side effects (i.e. increased pathogen susceptibility) of immunodrugs neutralizing IL-17 responses.

It was interesting to note that IL-17A and IL-17F and several genes known to be regulated by IL-17, e.g. CXCL1, CXCL2, CXCL5, CCL2, CCL7 and S100A9, were highly induced in the *S*. Typhimurium infected cecum mucosa, but that IL-17RA deficiency did not affect cytokine expression (Fig. 4). Several different explanations might account for this. i) A possible lack of IL-17RA expression in the cecum mucosa. This does not seem likely, as IL-17RA is expressed ubiquitously [30], [34]. This was verified for the duodenum, jejunum, ileum,cecum and colon tissues of naïve mice (Fig. 6A). In the inflamed cecum, *Il17ra* expression was even more pronounced (Fig. 6B). Furthermore, the disruption of IL-17RA signaling in enterocytes reduced the pathology of DSS-induced colitis [16] and IL-17A and -F are known to induce enterocyte expression of antimicrobial peptides and numerous pro-inflammatory cytokines. Thus, lack of *Il17ra* expression cannot account for our failure to detect a virulence phenotype in the gut mucosa of *Il17ra^−^* ^/*−*^ mice. ii) Insufficient levels of IL-17A/F induction. It is known that IL-17A and IL-17F differ significantly in terms of their threshold concentration required for inducing IL-17RA-mediated responses [30], [35], [36], [37]. The cytokine threshold concentration for IL-17RA responses in the cecum tissue is not known. Our data suggests that the IL-17A and -F levels in the cecum mucosa might simply not reach the required levels for inducing mucosal IL-17RA responses. On the other hand, the IL-17A/F induction was as pronounced as reported earlier for day 2 p.i. [1], [18]. iii) Timing of responses. One might argue that the 12 h p.i. time point might not allow sufficient time for IL-17 induced responses (Fig. 4 and 5). However, the expression analysis included several host genes known to be regulated by IL-17, e.g. CXCL1, CXCL2, CXCL5, CCL2, CCL7 and S100A9. All of them were strongly induced in the cecum mucosa at 12 h p.i. IL-17RA-deficiency did not significantly affect these responses. Thus, the expression of these genes does not require IL-17 signaling at 12 h p.i.. This does not exclude that IL-17 signals might contribute to sustained pro-inflammatory gene expression at later stages of the infection (day 2 p.i. [19]). iv) Cytokine redundancy. During infection IL-17A, can induce multiple pro-inflammatory cytokines, chemokines and antimicrobial effectors (reviewed in [33]). However, many other cytokines can do so, too and multiple signals, including IL-22 and IFNγ [19], [20], will drive tissue inflammation in a redundant fashion during the course of a real infection. Thus, disrupting just one of these cytokine-signaling pathways, as in *Il17ra^−/−^* mice, may not be sufficient for blunting the tissue-wide response. v. Receptor redundancy. Our study focused on IL-17RA and its ligands IL-17A and IL-17F. However, some studies suggest that IL-17RC homodimers can also bind and respond to IL-17F [30], [38]. Our IL-17F-neutralization experiments suggest that this type of IL-17F to IL-17RC signaling does not contribute significantly to mucosal inflammation. However, formally we cannot rule out that this signaling may contribute in some way. Future work will have to address these hypotheses.

We cannot exclude that IL-17A and -F may have important functions (besides acute mucosal responses) for immunity and defense. For example, these IL-17 cytokines may initiate adaptive immune responses or granulopoiesis in the bone marrow. These phenotypes have not been analyzed in the present study and would be an interesting topic for future research.

In conclusion, we found that deficiency in IL-17A or IL-17F signaling did not affect the initial phase of acute *Salmonella* enterocolitis in the streptomycin mouse model. This may indicate that the cytokine network eliciting mucosal inflammation in response to the *S*. Typhimurium infection is highly redundant or that other cytokines might be more important drivers of inflammation. Indeed, recent work has implicated a key role of caspase-1-mediated IL-1 and IL-18 responses in mucosal inflammation elicited by SopE [9]. However, this work also demonstrated that additional cytokines must be involved in the response to the wild type pathogen. Identifying these responses will be an important task for future work and is the key for the mechanistic understanding of the disease and for identifying novel targets for cure and/or prevention of the disease.
